# Unified ICH quantification and prognosis prediction in NCCT images using a multi-task interpretable network

**DOI:** 10.3389/fnins.2023.1118340

**Published:** 2023-03-14

**Authors:** Kai Gong, Qian Dai, Jiacheng Wang, Yingbin Zheng, Tao Shi, Jiaxing Yu, Jiangwang Chen, Shaohui Huang, Zhanxiang Wang

**Affiliations:** ^1^The First Affiliated Hospital of Xiamen University, Xiamen University, Xiamen, Fujian, China; ^2^Department of Computer Science, School of Informatics, Xiamen University, Xiamen, Fujian, China; ^3^Ningbo Medical Center Lihuili Hospital, Ningbo, Zhejiang, China

**Keywords:** intracerebral hematoma (ICH), Non-Contrast head Computed Tomography (NCCT), multi-task, ResNet, interpretability

## Abstract

With the recent development of deep learning, the regression, classification, and segmentation tasks of Computer-Aided Diagnosis (CAD) using Non-Contrast head Computed Tomography (NCCT) for spontaneous IntraCerebral Hematoma (ICH) have become popular in the field of emergency medicine. However, a few challenges such as time-consuming of ICH volume manual evaluation, excessive cost demanding patient-level predictions, and the requirement for high performance in both accuracy and interpretability remain. This paper proposes a multi-task framework consisting of upstream and downstream components to overcome these challenges. In the upstream, a weight-shared module is trained as a robust feature extractor that captures global features by performing multi-tasks (regression and classification). In the downstream, two heads are used for two different tasks (regression and classification). The final experimental results show that the multi-task framework has better performance than single-task framework. And it also reflects its good interpretability in the heatmap generated by Gradient-weighted Class Activation Mapping (Grad-CAM), which is a widely used model interpretation method, and will be presented in subsequent sections.

## 1. Introduction

Spontaneous IntraCerebral Hematoma (ICH) is characterized by high incidence, high disability, and high mortality, accounting for approximately 10–20% of all strokes (Raafat et al., 2020). The initial hematoma volume is the strongest predictor of mortality and functional outcomes (Beslow et al., 2014). Fast and precise evaluation of ICH volume through Non-Contrast Computed Tomography (NCCT) images is generally considered the first and the most critical step to making further clinical decisions including medical and surgical options. As the evaluation of ICH volume is relatively a time-consuming process, head NCCT reports usually provide only a general description of ICH location and shape, whereas the ICH volume is not calculated. In most cases, the ICH volume is assessed manually by clinicians such as neurosurgeons or neurology physicians, using various methods including ABC/2, ABC/2.4, ABC/3,1/2Sh, and 2/3Sh. Among them, ABC/2, also known as the Tada formula, is the most used one. For further explanation, A is the maximum axial hematoma diameter, B is the maximum axial diameter perpendicular to A on the same slice, and C is the vertical diameter of the hematoma (Beslow et al., 2010). The reliability of the ABC/2 formula is shown good correlation with computerized ICH volume measurements for small and uniformly shaped ICHs, but the accuracy can be affected by observer variability and imprecision (Oge et al., 2021). The method is prone to overestimate ICH volume by approximately 20% and even misestimate more for large and irregularly shaped hemorrhages (Patel et al., 2019). The 3D slicer software (http://www.slicer.org) provides a free open source software platform for biomedical research. It can identify hematoma pixels based on CT images and reconstruct blood clots in a three-dimensional manner, which is free from restriction by hematoma morphology and bleeding sites. Compared with manual methods, 3D slicer method is considered as a more stable and capable method of high precision for the volume calculation of most hematomas and is gradually accepted as an effective measurement method clinically (Xu et al., 2014; Chen et al., 2020; Gong et al., 2021).

Newly automatic, versatile, easily deployable, and accurate hematoma volume assessment tools are highly needed giving their clinical significance. Numerous attempts have been conducted to develop 3D slicer as well as other computer-assisted automated tools for hematoma volume evaluation (Huttner et al., 2006; Wang et al., 2009; Zhao et al., 2009; Yang et al., 2013; Xu et al., 2014), but the methods have not been fully automated yet, and still require a significant amount of decisions to be made by researchers, which remains a challenging problem.

With the recent growth of deep learning, the evaluation methods of hematomas with deep learning-based techniques have been advanced dramatically, including the ResNet model, the DenseNet model, and the H-DenseUNet model, see Zhao et al. (2021), Mantas (2020), Dawud et al. (2019), Zhou et al. (2022), and Gou and He (2021).

For the evaluation of intracranial hemorrhage volume, the deep learning method has been proved to be superior to the clinical method in stability and accuracy many times (Freeman et al., 2008; Sharrock et al., 2022). Most quantitative assessments of ICH volume are based on the segmentation or classification of ICH areas. Phaphuangwittayakul et al. (2022) propose a quantitative assessment algorithm to automatically measure both thickness and volume *via* the 3D shape mask combined with the output probabilities of the classification network. Xu et al. (2021) realize hematoma segmentation and volume evaluation by Dense-Unet (Cai et al., 2020; Sharrock et al., 2021), and is based on V-Net architecture. Much literature focuses on developing better neural network architectures and training strategies to optimize ICH segmentation, on which post-processing can be performed to automatically measure the volume of intracerebral hemorrhage. In this paper, we do not have the segmentation mask of the cerebral hemorrhage area. Instead, we have the measurement results of the volume of cerebral hemorrhage by multiple doctors. On the basis of training on this, we expect the model to be able to locate the bleeding location and even mark the bleeding area, which is what we intend to continue to study in the future.

This paper describes a lightweight multi-task learning framework firstly, which is specifically designed to identify and evaluate ICH hematoma volumes by training a large number of NCCT images collected from 258 patients with ICH. Then, based on the assessment of ICH hematoma volumes, further prognosis analysis of intracerebral patients through the multi-task framework is discussed. Finally, satisfactory results are obtained for both tasks. For the evaluation of ICH hematoma volumes, the effect of this model is even better than the assessments of some clinicians. To further improve the interpretability of the model, Gradient-weighted Class Activation Mapping (Grad-CAM) is utilized to visualize the prediction of the model, which focuses well on the bleeding area and provides a reliable basis for the predictive output of the model. This can make a lot of sense for clinical application.

## 2. Related work

### 2.1. Deep learning in brain computerized tomography images

The application of neuroimaging technology plays an important role in the diagnosis and treatment of cerebrovascular diseases and is an indispensable auxiliary diagnostic tool. As the main imaging methods of the brain, computerized tomography (CT) and Magnetic Resonance Imaging (MRI) are widely used in clinical practice. Among them, CT examination is the first choice to find most brain diseases, including congenital brain development intracranial abnormalities, brain tumors, cerebral hemorrhage, and so on. The major advantages of CT images after reconstruction for medical image analysis are high density and clear image; it can assist clinicians to master the brain structure and abnormalities within the brain tissues easily (Padma Nanthagopal and Sukanesh Rajamony, 2013; Sachdeva et al., 2013; Vidyarthi and Mittal, 2014). Researchers utilize different automated approaches for brain disease detection and type classification through brain radio images since medical images can be scanned and uploaded to computers with a fine-resolution. For a long time in the past, Support Vector Machine (SVM) and Neural Networks (NN) techniques have been widely used due to their stable and good performance (Pan et al., 2015). But in recent years, due to the improvement of equipment computing power, Deep Learning (DL) models have created an exciting new trend in the field of machine learning, because deep architecture can efficiently represent more complex relationships without requiring a large number of nodes like traditional machine learning, such as SVM and K-Nearest Neighbor (K-NN). With the vigorous development of these technologies, they have become advanced technologies in different medical and health fields, such as bioinformatics, medical informatics, and medical image analysis. Among all kinds of deep architectures, the Convolutional Neural Network (CNN) is undoubtedly the most commonly used architecture now, which can use the convolution kernel to realize complex operations such as feature extraction (Pan et al., 2015; Rav̀ı et al., 2016; Litjens et al., 2017). Usually, CNN is designed for image recognition tasks. The image is first processed by multiple convolutional layers (each convolutional layer is followed by a Rectified Linear Unit (ReLU) layer and a pooling layer), then input to the fully connected layer and the ReLU layer. Finally, the output layer produces the prediction of class probabilities. Although the CNN architecture does not require manual feature extraction compared with traditional machine learning methods, it is very difficult to train a CNN model from scratch, which requires a large number of labeled data sets for adequate learning, especially for tasks such as classification and regression: the data volume requirements are relatively large. Moreover, for processing a large number of filters, such as large-scale medical 3D images such as 256 × 256 × 32, the hardware requirements are very high (Ben Ahmed et al., 2017).

The contribution of this paper is to apply deep learning to the prediction of intracranial hemorrhage. Due to the correlation between intracranial hemorrhage and prognosis survival, this paper further proposes a lightweight multi-task learning framework based on the prediction of ICH hematoma volume. The aim is to further effectively predict the prognosis of patients according to the relevant features of the blood loss learned by the model.

### 2.2. Interpretability

Deep learning is a multi-layer representation learning method built with simple nonlinear modules that transform the previous layer representation into a higher-level, more abstract representation, so that it is able to detect increasingly abstract features. Therefore, a large number of features are also generated in the process of generating the prediction results, which makes the information very compact, especially in the deep layers. It's hard to explain this process, so we don't know why the neural network makes this prediction. This is called the “black box” problem of deep neural networks (Castelvecchi, 2016). Indeed, they are capable of producing extremely accurate predictions, but how can predictions based on features beyond comprehension be reliable? It is a challenging problem.

In medical images, the interpretability of the model is even more important: it determines whether the clinicians can trust and accept the model predictions which could be critical in clinical applications. Interpreting the predictions of deep models is hard without appropriate techniques, but a range of approaches has emerged over the past few years. One example is to show the texture of imaging features at a single layer to examine the hierarchical process of learning (Zeiler et al., 2011). In addition, numerous researches and explorations have been carried out on generating meaningful heatmaps in order to highlight the importance of individual pixel regions in the input image for the final CNN prediction. Many techniques have demonstrated the feasibility of generating heatmaps, including strategies using deconvolution (Zeiler and Fergus, 2014), layer-wise relevance propagation (Samek et al., 2016), and saliency map construction (Simonyan et al., 2013; Ghorbani et al., 2020; Thomas et al., 2020). However, these methods still have certain shortcomings, such as being susceptible to noise and artifacts, lacking sensitivity to input disturbances and qualitative criteria for evaluating the quality of back propagation (Samek et al., 2016; Smilkov et al., 2017; Ghorbani et al., 2020). Class Activation Mapping (CAM) serves as a better alternative approach. But in its most basic implementation, CAM requires adding a pooling layer to the target model, limiting the interpretation to only one specific layer (Zhou et al., 2016). Recently, two extended versions of CAM have emerged, namely, Gradient-weighted Class Activation Mapping (Grad-CAM) (Selvaraju et al., 2017), and Grad-CAM++ (Chattopadhay et al., 2018). Compared with CAM, both of them can interpret arbitrary layers of CNN without any architectural modification, thus increasing the flexibility of the use.

Grad-CAM is one of the most widely used techniques for prediction interpretation; it is chosen in this paper to interpret the prediction of the model and to increase the credibility of the model so that it has good interpretability in clinical application.

## 3. Method

To obtain the IntraCerebral Hemorrhage (ICH) volume and provide interpretative decision details, we propose a novel multi-task learning framework to take the complementary advantages of ICH regression and classification. Besides improving the accuracy of both tasks, the fusion is also able to enhance the interpretability which is of great significance for the clinical application.

### 3.1. Overall architecture

As described above, the proposed network adopts the multi-task learning manner allowing the model to learn from different annotations. It can not only boost knowledge extraction by sharing the convolutional parameters but also can accelerate the learning program by calculating gradients from multiple branches. The motivation is the correlation between ICH regression and classification tasks. The research in Brott et al. (1997) shows that the increase of bleeding volume in the early stage of ICH is an important factor determining the prognosis of ICH patients. Unfortunately, the mortality rate of ICH is about 40% per month, with 61–88% of survivors having degrees of residual disability. In this context, the hematoma size is a key character for prognosis predictions. Therefore, it is more accurate and acceptable to predict the prognosis of patients based on a certain accurate assessment of the bleeding amount.

**Figure 1:**
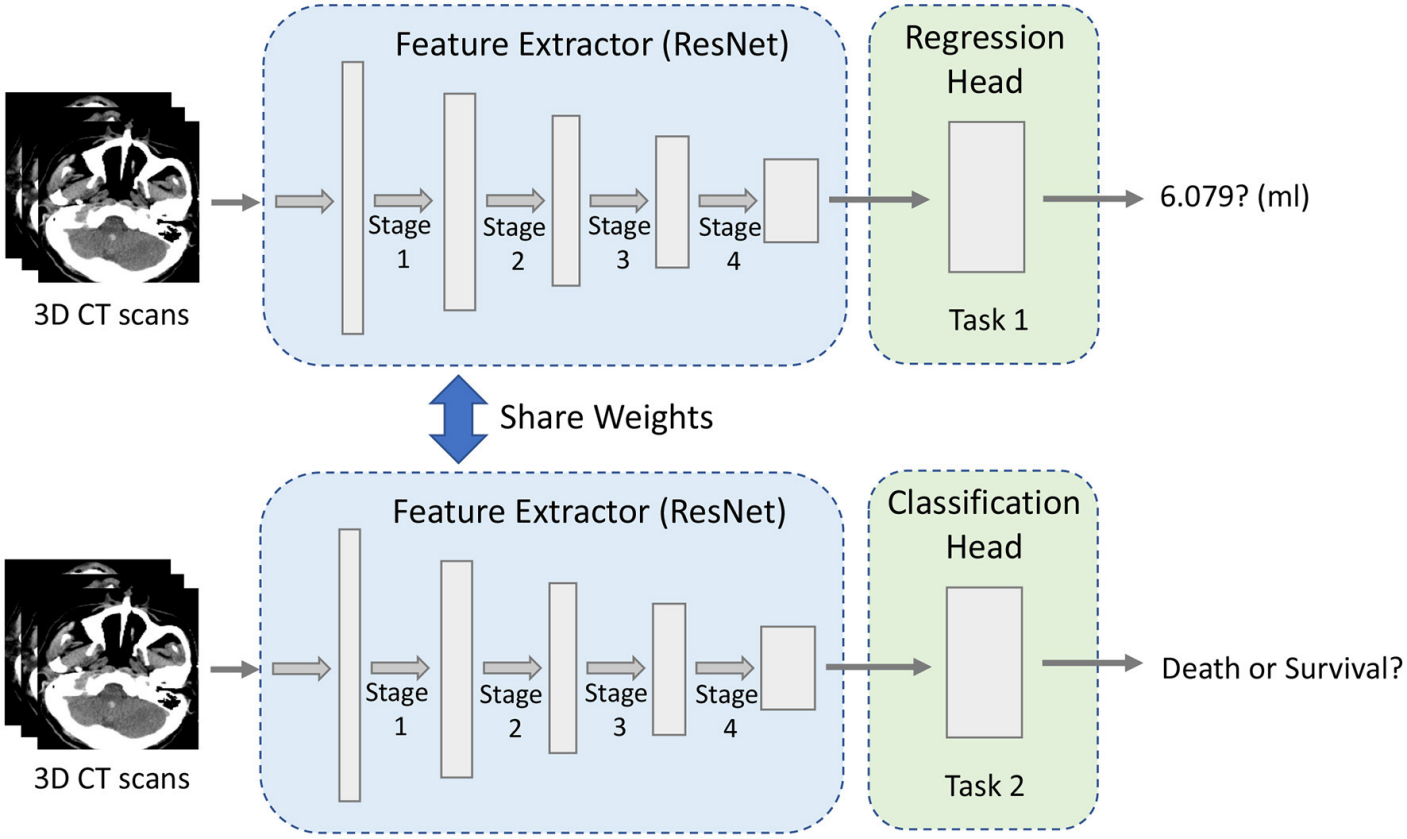
The overview of our multi-task framework.

Our multi-task CNN architecture is proposed to jointly learn from both tasks, whose details are shown in Figure 1. The major components of the proposed network include a shared module for brain image feature extraction and two heads corresponding to different tasks. The algorithms for regression and classification tasks are built on ResNet deep neural network architecture (He et al., 2016), which includes the residual information extracted from the previous layer and mitigated the adverse performance by using a large number of layers.

### 3.2. Efficient feature extraction

Experience suggests that the multi-task model often achieves better results than the single-task model. Compared to single-task learning, multi-task learning has the following advantages: shared encoder can reduce parameter amounts and save computation, associated tasks can improve each other's performance by sharing information and complementing each other. Also, it is helpful for the bottom-sharing encoder to learn the common feature representation.

The essence of multi-task learning lies in sharing the presentation layer and making the tasks interact with each other when the correlation between the predicted goals is relatively high.^1^ Consequently, the parameter sharing layer will not bring too much loss, the parameter sharing layer can strengthen the parameter sharing, multiple target models can be trained jointly, reduce the parameter scale of the model and prevent the model from overfitting. Our final experimental results also fully demonstrate the effectiveness of applying multi-task learning to our tasks.

For multi-task learning, learning a general feature representation is very important for the model. As shown in Figure 1, the robust feature extraction module is designed to extract features of a 3D image and obtain a high-quality feature vector, which is used for downstream tasks. The module is based on 3D ResNet34 (He et al., 2016) because the residual structure effectively solves the problem of model degradation due to its depth, skip connection and also enhances the information transfer between the upper and lower layers.

Given a 3D CT image of 224 × 224 × 32 pixel size, the features are extracted by one stem block and four stages of ResNet (see Figure 1). Specifically, the first layer is a convolutional layer with a 7 × 7 × 7 kernel size and 64 filters. The pooling layer is set after that to decrease the image's size and save the computation cost. The max pooling layer with a kernel size of 3 × 3 × 3 and stride 2 is used here empirically. The second, third, fourth, and fifth phases are made up of multiple convolutional operations, corresponding to the four stages in Figure 1. The first is a convolution layer with a filter size of 3 × 3 × 3 and the rest of the three modules are stacked on the top of each layer. The second is a convolution layer with the filter size of 3 × 3 × 3 and there are four residual blocks. Each convolutional layer in the network applies zero-padding, after each convolutional layer, a batch normalization layer (Ioffe and Szegedy, 2015) is applied to speed up model training and convergence. To allow the application to images of arbitrary size, 3D feature maps are not flattened but dense layers are implemented as convolutions with the size of 1 × 1 × 1 (Long et al., 2015).

After the entire feature extraction process, the final extracted feature map is obtained. The network has two output heads, one for ICH volume regression and another one concerns the classification of the prognosis. Finally, the features extracted by the last layer of the module are fed to each task-specific head.

### 3.3. Regression head

The first task-specific head for regression only has an average pooling layer and one fully connected layer. The final output is a scalar referring to the amount of ICH volume predicted by the model. This regression task is performed to predict the IntraCerebral Hemorrhage (ICH) volume in each 3D CT scan, task-specific head module to find the most intensive crux features of ICH. The regression loss is defined as the Mean Square Error Loss (MSE Loss) defined by *L*_*reg*_:


(1) $$L_{reg}=\frac{1}{N}\sum_{i=1}^{N}{\left({\hat{y}}_{reg_{i}}-y_{reg_{i}}\right)}^{2},$$

Where $\hat{y}$_*re*_*g*__*i*__ is the predicted output of the regression head, and *N* is the number of CT images.

### 3.4. Classification head

The second task-specific head for classification has a simple linear classifier which consists of a global average pooling, a linear layer, and a ReLU activation function. Finally, the softmax function is applied to the output to obtain the probability of the model prediction, which is between 0 and 1. Generally, we use 0.5 as the classification decision threshold: a probability greater than 0.5 is fixed to 1, which means that the class belongs to, whereas a probability less than 0.5 is fixed to 0, meaning that the class does not belong to. The upstream framework is the same and shares the weight of the feature extraction module of the regression task. The classification loss is defined as the Binary Cross-Entropy Loss (BCE Loss) called *L*_*cls*_:


(2) $$\begin{array}{l} L_{cls}=\frac{1}{N}\cdot \overset{N}{\underset{i=1}{\sum }}\left[y_{cls}\cdot \log\left({\hat{y}}_{cls_{i}}\right)+\left(1-y_{cls_{i}}\right)\cdot \log\left(1-{\hat{y}}_{cls_{i}}\right)\right], \end{array}$$

Where $\hat{y}$_*cl*_*s*__*i*__ is the predicted output of the classification head, and *N* represents the number of CT images.

For our multi-task framework, the feature extractor is shared and the two heads fit the outputs of the two tasks separately. Therefore, the loss of multi-task framework is a combination of the losses of two tasks. Through the simple weighting of loss, the network weights are updated to optimize.

### 3.5. Interpretive design

Traditional deep learning applications lack interpretability and thus faces limitation in clinical practice. We propose to utilize the advanced Grad-CAM to explore how the results are obtained. Specifically, class weights derived in Grad-CAM used the equation:


(3) $$\begin{array}{l} w_{k}^{c}=\frac{1}{Z}\underset{i}{\sum }\underset{j}{\sum }\frac{\partial y^{c}}{\partial A_{ij}^{k}} \end{array}$$

Where *Z* denotes the total number of elements in a feature map *A*, $A_{ij}^{k}$ represents the data of the feature map in channel *k*, and the coordinate is *ij*, *y*^*c*^ denotes the score predicted by the network for class *c*.

This equation is backpropagated through the prediction score of class *c* to obtain the gradient information that is backpropagated back to feature layer *A*, namely $w_{k}^{c}$, which represents the importance of each channel of feature map *A*, here for class *c*. The higher the value, the greater the contribution, and the more important it is considered by the model. Finally, weighted summation is performed and the final Grad-CAM heatmap is obtained by ReLU activation function. Consequently, the heatmap generation uses the formula:


(4) $$\begin{array}{l} L_{Grad-CAM}^{c}=ReLU\left(\underset{k}{\sum }w_{k}^{c}A^{k}\right), \end{array}$$

Where the ReLU (Rectified Linear Unit) function allows to evaluate features with positive impact only. In this paper, we use the last convolutional layer in computing the weights as suggested previously.

## 4. Experiments

### 4.1. Ethics approval and consent to participate

The study is undertaken in compliance with the principles of the Declaration of Helsinki and is approved by the ethics committees of the First Affiliated Hospital of Xiamen University (Approved ID: SL-2020KY034). All individual patient data used for the analysis are collected by providers after obtaining appropriate consent and agreements.

### 4.2. Data collection

This study involves 258 patients with spontaneous intracerebral hematoma (ICH) admitted to the First Affiliated Hospital of Xiamen University between April 2017 and February 2019 to develop the prognosis model. These axial brain Computed Tomography (CT) scans, taken on admission using a Philips Brilliance 64-row spiral CT scanner are exported from the Department of Neurosurgery through Picture Archiving and Communication Systems (PACS) and stored in Digital Imaging and Communications in Medicine (DICOM) format.

### 4.3. Data preprocessing

To evaluate the effectiveness of the developed network, we collect a new ICH dataset with 258 patients, of which 227 patients survived and 31 patients dead. All patients are given a table with the patient's name and measurements of the ICH volume (mililiter, ml) from eight professional neurosurgeons (with 2, 3, 3, 4, 4, 8, 15, and 20 years of experience) and the 3D slicer software, which is considered the gold standard.

**Figure 2:**
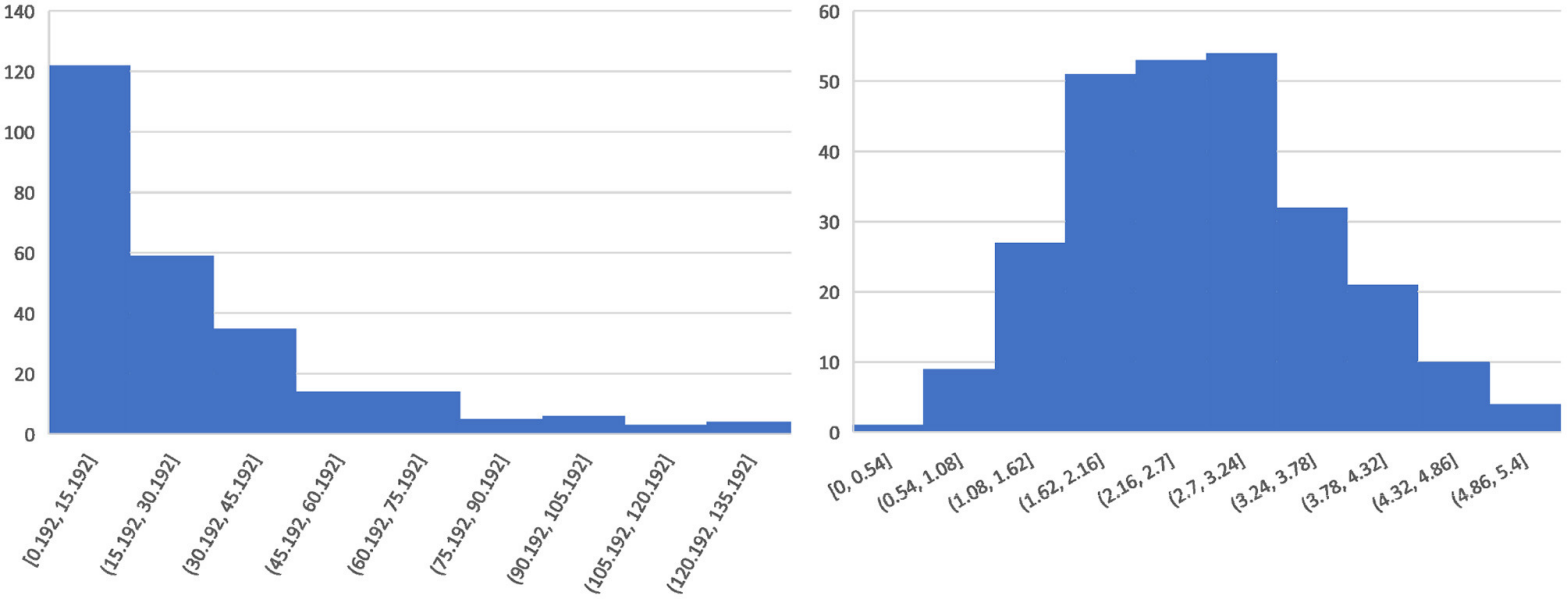
Histogram of the blood loss distribution.

For the regression labels, because of the uneven distribution of ICH volume (shown in Figure 2 left), we calculate the third root of the ICH volume and convert it to the ICH size (milimeter, mm), which is more balanced in distribution (shown in Figure 2 right) and more beneficial to model training. For the final prediction results of the regression model, we perform post-processing and then convert them into ICH volumes for further analysis and comparisons.

Considering the intensity range of brain characteristics (Fosbinder and Orth, 2011), we apply a brain window that clips [40, 110] Hounsfield units (HU) and normalized the input to [0, 1]. After cutting out the blank part of the brain in the image, the image size is resized down to 32 × 224 × 224 by linear interpolation. Since ICH can occur anywhere in the brain with multiple subtypes simultaneously, we do not crop the images and don't use patch images for training, which may lead to unstable results and false positives (Hu et al., 2011).

### 4.4. Evaluation metrics

For the regression task, we choose the following three indicators to evaluate, which include Mean Square Error (MSE), Mean Absolute Error (MAE), and Mean Absolute Percentage Error (MAPE). And MAPE is the relative error, which is the percentage of absolute error and truth. For classification task, three widely-used metrics are used for quantitative analysis, which are accuracy, ROC curve, and area under the curve(AUC).

### 4.5. Implementation details

Our network is implemented on Pytorch (Paszke et al., 2019) and trains using SGD (Robbins and Monro, 1951) with 300 epochs, an initial learning rate of 1 × 10^−4^, a momentum of 0.9, and a weight decay of 5 × 10^−4^. The whole architecture is trained on one GeForce RTX 3080 Ti GPU, and each GPU has a batch size of 8. For a fair comparison, our method and all models follow the same training settings.

### 4.6. Results

The performance of our proposed approach for regression and classification tasks is reported in Tables 1, 2, respectively.

**Table 1:** Quantitative comparisons for the effectiveness of regression model.

**Methods**	**MAE**	**MSE**	**MAPE(%)**
Clinician 1	5.21	65.93	28.78
Clinician 2	6.14	84.10	38.50
clinician 3	4.46	39.15	38.68
Clinician 4	4.77	50.05	34.13
Clinician 5	4.88	72.04	29.60
Clinician 6	5.30	56.08	44.65
Clinician 7	3.86	38.84	20.14
Clinician 8	4.02	40.98	24.42
X3D (Feichtenhofer, 2020)	18.28	490.19	99.98
R(2+1)D (Tran et al., 2018)	7.09	80.02	45.59
P3D (Qiu et al., 2017)	10.24	143.54	171.18
TSM (Lin et al., 2019)	9.51	130.69	160.93
Times Former (Bertasius et al., 2021)	10.49	158.13	171.09
TIN-ResNet-18 (Shao et al., 2020)	7.90	107.52	110.68
TIN-ResNet-34 (Shao et al., 2020)	9.71	134.73	159.54
TIN-ResNet-50 (Shao et al., 2020)	10.87	170.85	184.58
Uniformizing-3D (Zunair et al., 2020)	7.87	118.06	102.38
Multi-task (ours)	4.91	52.65	43.00

The first eight lines denote the measurements of eight clinicians.

**Table 2:** Quantitative comparisions of the effectiveness of multi-task framework.

**Methods**	**ACC (Accuracy)**	**AUC**
ResNet (single-task)	88.68	87.94
ResNet (multi-task, ours)	**94.34**	**93.62**

“Single-task” denotes the classification model train from scratch.

Bold values indicate the optimal result.

To verify the effectiveness and feasibility of our proposed framework, Table 1 selects five existing methods widely used for 3D image classification tasks for comparison, including X3D (Feichtenhofer, 2020), R(2+1)D (Tran et al., 2018), P3D (Qiu et al., 2017), TSM (Lin et al., 2019), Times Former (Bertasius et al., 2021), TIN (Shao et al., 2020) with backbone of ResNet-18, 34, 50 and Uniformizing-3D (Zunair et al., 2020), which is a 3D CNN for CT scans . For providing a fair comparison, we obtain the final classification results on the official implementations of these compared methods. We train these models on our dataset and set the same experimental parameters as ours. As shown in Table 1, the model trained by deep learning performs well. The method we choose based on ResNet architecture achieves the best results and is even more suitable for the tasks of regression and classification of 3D brain CT images, with MAE reaching 4.91, the MSE reaching 51.92, and MAPE reaching 43.00%. Compared to the evaluation results of eight clinicians (first eight rows in Table 1), our method also has competitive performance, outperforming the evaluation results of three clinicians (clinicians 1, 2, 6), while also having better interpretability.

**Figure 3:**
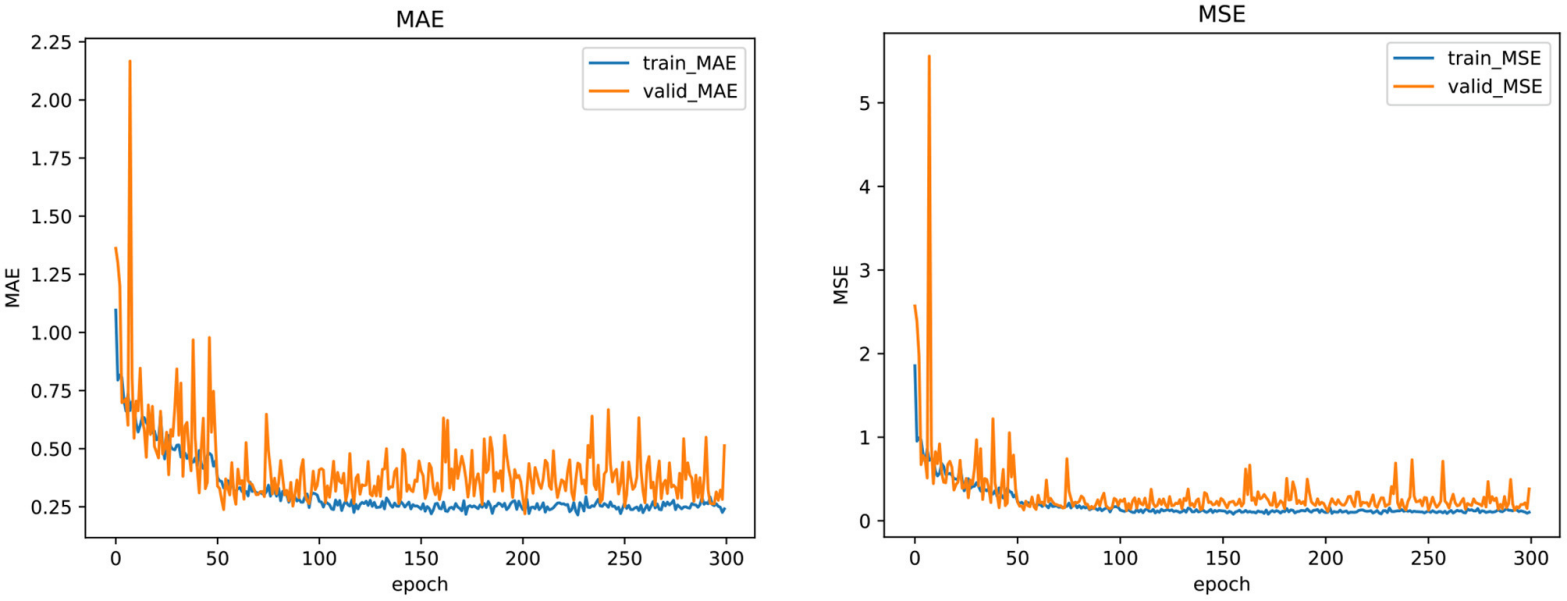
The training curve of the regression loss in our multi-task framework.

To further show the stability and effectiveness of our model training, we plot the curve of the indicator change during the model training and validation process, including MAE and MSE, as shown in Figure 3. From the figure, we can see that whether in the training or validation phase, the MAE and MSE loss functions of our model gradually decrease and converge to be stable in 300 epochs, which indicates that the model obtained by our training is stable and reliable. The training of other models also shows a similar trend.

The results of the classification task shown in Table 2 demonstrate the effectiveness of the multi-task network. Compared with the single-task model, that is, trained from scratch, the performance of the multi-task model is also greatly improved due to the shared encoder parameters, with accuracy improved by 5.66% and AUC improved by 5.68%, and both reached more than 90%.

**Figure 4:**
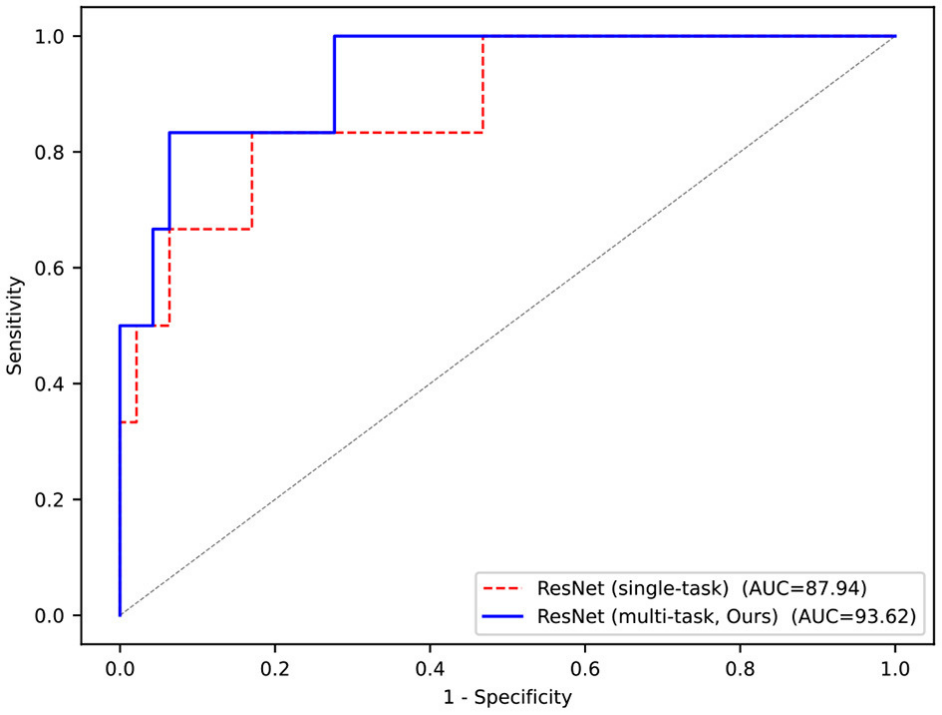
ROC curves of our multi-task framework and compared method.

More visually, Figure 4 shows the ROC curves of our multi-task model and single-task model. The performance of the multi-task model is better than others, and the area of AUC remains over 90%, which fully demonstrates its effectiveness.

### 4.7. Interpretability

To enhance the interpretability of the model for the prediction of ICH volume, we visualize the output of the last convolutional layer of the regression model. As mentioned earlier, we introduce Grad-CAM (Gradient-weighted Class Activation Mapping) (Selvaraju et al., 2017) to generate heatmaps, which show the region of interest of the model.

**Figure 5:**
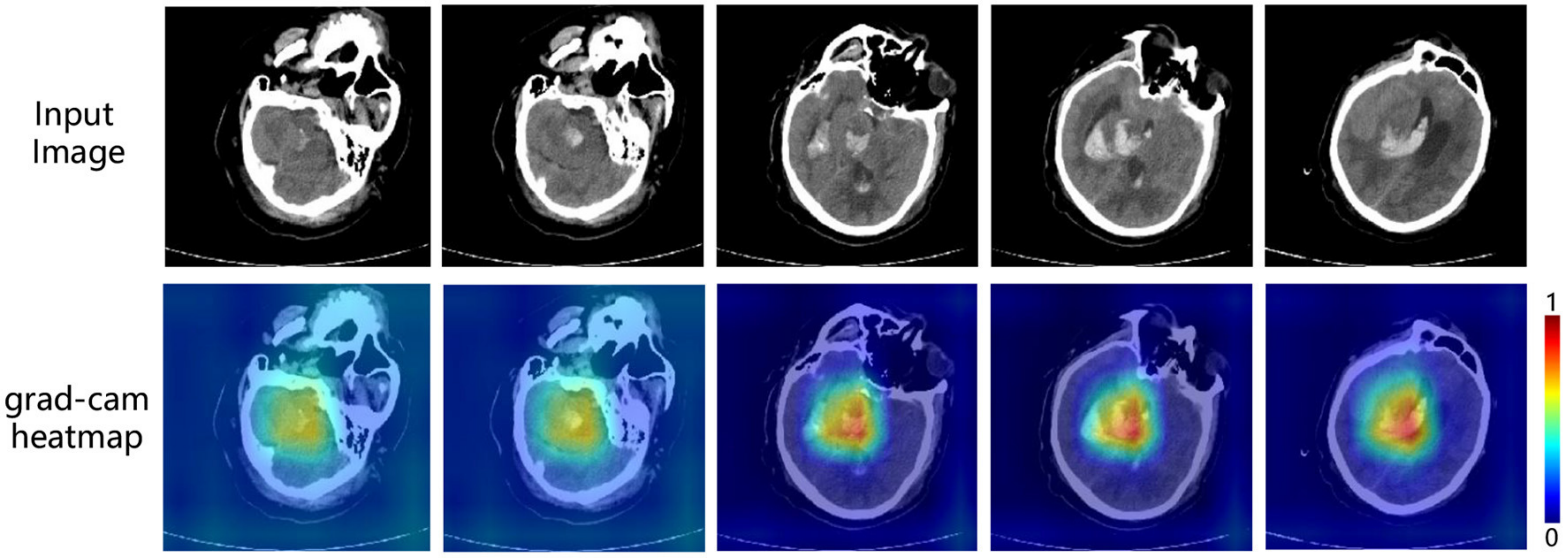
Interpretable visualization of our multi-task framework. The images are five slices taken from a 3D CT scans at medium intervals (*t* = 5).

In the heatmap, the brighter the color is, the more attention the model pays to. As shown in Figure 5, the highlighted areas are concentrated in certain parts of the brain with some white spots. And according to our confirmation with a professional clinician, this is exactly the location of the cerebral hemorrhage in the image. Therefore, it is reasonable to say that in the prediction of ICH volume, our model accurately locates the location of bleeding, to make the prediction, and the result is acceptable to clinicians. At the same time, it also provides a basis for subsequent prognostic analysis tasks, as the ICH volume is a key factor affecting patient mortality.

## 5. Discussion

### 5.1. Clinical significance for proposed algorithm

Stroke is the second-leading cause of death, and the third-leading cause of death and disability combined in the world (Feigin et al., 2021). Intracerebral hematoma (ICH) is a common and fatal subtype of stroke, which is characterized by rupture of arterial blood vessels and formation of hematoma within the brain parenchyma, such as the basal ganglia (Zhang et al., 2020; Kinoshita et al., 2021). The formation of a large hematoma compresses the surrounding brain tissue, resulting in neurological deterioration, intracranial hypertension, global cerebral ischemia, and brain herniation, which can affect vital autonomic structures (Yu et al., 2020). The mortality rate of ICH is about 40% per month, with 61–88% of survivors having high degrees of residual disability (Van Asch et al., 2010). Management of increased intracranial pressure is the mainstay of ICH treatment in the acute phase (Kase and Hanley, 2021). Patients with excessive bleeding and coma often require surgical treatment to relieve the hematoma, brain tissue compression, and secondary brain injury (Lin et al., 2014). However, as an invasive process, iatrogenic injury is inevitable during hematoma evacuation, which means the accurate assessments of the patients' condition and appropriate selections for surgical interventions are critically important. The hematoma size is a key character for prognosis predictions and clinical decision making. Supratentorial hematomas larger than 30 ml (Kerebel et al., 2013; Huang et al., 2021) and infratentorial hematomas larger than 15 ml (de Oliveira Manoel, 2020) are often considered to result in poor prognosis and tend to require surgical evacuation. Thus, the accuracy of volume evaluation for ICH patients has significant clinical implications.

There are mainly four kinds of methods to evaluate ICH volume, including the mathematical formula method, tool measurement method, CT machine measurement method, and software method (Chen et al., 2020). Among them, Tada formula, a kind of formula method, is the most commonly used one. However, the accuracy of Tada formula can be affected by both hematoma shape and volume, resulting in imprecise disease evaluation and potential improper treatments (Gong et al., 2021). 3D Slicer is shown to be more precise than most methods (Chen et al., 2020), but the modeling process is relatively time-consuming for clinicians. With the development of deep learning techniques, artificial intelligence has provided promising results in the medical field and may help with medical image interpretation and prognosis prediction, offering more reliable results than manual decisions by clinicians. Several previous studies have used deep learning-based techniques to evaluate ICH volumes (Roh et al., 2019), but there are still some shortcomings. For instance, some studies use the manually estimated hematoma volume as the label to train the model, which is not accurate enough to evaluate the performance of the model because the relatively large error of manual estimation leads to the inaccuracy of the label. In addition, it does not take the problem of model interpretation into account.

For this reason, this study uses the results of 3D slicer as labels to improve the accuracy of model training and evaluation. At the same time, in addition to achieving competitive accuracy with clinicians, our model has good interpretability and is easier to be understood and accepted by clinicians. Intuitively, we attribute the accuracy of model prediction to the fact that the heatmap shows that it focuses on the information related to the bleeding area, which is in turn the target of the model being explained. This demonstrates the accuracy of the effect of our model, and its concerns are consistent with those of professional physicians. Furthermore, the evaluation of hematoma volume to predict patient prognosis is not accurate enough because the ICH density, shape, location, the middle line deviation, the effacement of Sylvian fissure and perimesencephalic cisterns (Kim, 2012) are all related to the prognosis of ICH. Therefore, this study uses the complete CT images as the training objects and does not mark a specific region of interest, so that the results are more reasonable and robust.

### 5.2. Limitations and further directions

Our experimental results prove the effectiveness of our proposed framework well, but to better apply it clinically, we should test it on more data. This is also the current limitation of our method. First, our data are collected from a single center and the result should be further validated by data from multiple centers in terms of predicting prognosis due to differences in treatment levels. Second, given the variable performance of ICH, the sample size is still needed to be expanded, including hemotomas of different sizes, locations, shapes, and densities. Third, cerebral hemorrhage is a dynamic process, and a single NCCT can only be used to evaluate static intracranial conditions. Therefore, subsequent studies can use different concurrent images as time series for prognostic evaluation. Fourth, besides the radiological features, the clinical features including age, the severity of other underlying diseases, the GCS coma score, etc. are all associated with prognosis (Wang et al., 2014). Using multimodal data may further advance the performance of such clinical prediction models.

## 6. Conclusion

We introduce a multi-task framework for ICH volume (regression) and patient prognosis (classification) prediction in NCCT. First, we design a robust feature extractor through the ICH volume prediction task, which effectively locates and focuses on ICH regions. This is effectively demonstrated in the Grad-CAM. By exploring the relationship between ICH volume and patient prognosis, we find that the effectiveness of feature extraction on the former task affected the performance on the latter task. A feature extractor that can well locate the hemorrhage area and pay attention to the hematoma, which is very beneficial for the subsequent task of prognostic analysis. The experimental results also prove it well. Compared with existing methods, our framework achieves impressive results on both tasks, and its reliable interpretability also makes it possible for clinical application.

## Data availability statement

The raw data supporting the conclusions of this article will be made available by the authors, without undue reservation.

## Author contributions

KG, ZW, and SH contributed to the study concept and design. KG and YZ collected the data. KG, QD, and JW performed the statistical analysis and wrote the manuscript. YZ, TS, JY, and JC revised the manuscript. All authors contributed to the article and agreed to the submitted version.

## Footnotes

^1^In our task, ICH volume regression and patient prognosis classification based on brain CT images. The underlying features of both should be similar, both at the bleeding site in the brain.
